# Gastroparesis-Related Symptoms in Patients With Type 2 Diabetes Mellitus: Early Detection, Risk Factors, and Prevalence

**DOI:** 10.7759/cureus.35787

**Published:** 2023-03-05

**Authors:** Shoaib Asghar, Sohaib Asghar, Salman Shahid, Hasnain Sajjad, Jamal Abdul Nasir, Muhammad Usman

**Affiliations:** 1 Internal Medicine, Shaikh Zayed Medical College and Hospital, Rahim Yar Khan, PAK; 2 Gastroenterology, Glan Clwyd Hospital, Betsi Cadwaladr University Health Board, Rhyl, GBR; 3 Internal Medicine, Bedfordshire Hospitals NHS Foundation Trust, Bedford, GBR; 4 Pediatrics, City Hospital Multan, Multan, PAK; 5 Emergency Medicine, Queen Elizabeth Hospital, London, GBR; 6 Emergency Department, Queen Elizabeth University Hospital, Glasgow, GBR

**Keywords:** patient assessment of gastrointestinal disorders-symptom severity index (pagi-sym), hyperglycemia, glycated hemoglobin (hba1c), gastroparesis cardinal symptom index (gcsi), body mass index (bmi), type 2 diabetes mellitus (t2dm), gastroparesis

## Abstract

Background

The symptoms of gastroparesis, such as bloating, postprandial fullness, early satiety, nausea, and abdominal discomfort, progressively worsen the quality of life of the affected individuals. The diagnosis is established on the assessment of gastric function that confirms delayed gastric emptying in the absence of structural etiologies. This study aimed to detect gastroparesis-related clinical symptoms early in patients with type 2 diabetes mellitus (T2DM), investigate the concomitant risk factors, and evaluate the prevalence.

Methodology

This study was conducted at the Department of Medicine and Diabetes Outdoor Clinic of Sheikh Zayed Hospital, Rahim Yar Khan from February 13, 2022, to February 11, 2023. The study involved 175 patients with T2DM who reported gastroparesis-related symptoms. The demographic and clinical characteristics, symptom severity, complications, related risk factors, duration of disease, medications, body mass index (BMI), fasting plasma glucose, and glycated hemoglobin (HbA1C) levels were assessed. The severity of diabetic gastroparesis was established using the disease-specific Patient Assessment of Gastrointestinal Disorders-Symptom Severity Index (PAGI-SYM) and the Gastroparesis Cardinal Symptom Index (GCSI). The five-point scale of the PAGI-SYM and the four-degree severity scores of GCSI were assessed. Neuropathy disability scores and motor evacuation functions were analyzed. Data were analyzed from these questionnaires, special proforma, and patient interviews.

Results

The clinical features of diabetic gastroparesis were observed in 44% of T2DM patients with mild-grade gastroparesis in 38 (21.7%), moderate in 30 (17.1%), and severe-grade gastroparesis-related symptoms in nine (5.2%) patients. The main manifestations were early satiety (45.1%), stomach fullness (44.5%), bloating (38.3%), and nausea (33.1%). Diabetic gastroparesis symptoms were considerably linked to disease duration of more than 10 years (p = 0.02), high HbA1c (p = 0.001), increased fasting blood glucose (p = 0.003), polyneuropathy, cigarette smoking, and history of comorbid conditions (p = 0.009). Obesity and the female gender were the forecasters of the manifestation of at least one cardinal gastroparesis symptom.

Conclusions

Gastric emptying is significant in the pathogenesis of gastroparesis-related symptoms. Disease duration of more than 10 years, poor glycemic control with hyperglycemia, high HbA1C, polyneuropathy, and cigarette smoking must be considered as predictors for early detection and risk factors for the advancement of gastroparesis in T2DM. Gastroparesis-related common symptoms of early satiety, bloating, and stomach fullness were considerably linked to the additional risk factors of hypercholesteremia, chronic microvascular complications, concomitant cardiovascular diseases, and a positive family history of diabetes mellitus. There was no relationship between BMI, age, types of treatment, and the degree of gastroparesis severity. The prevalence and severity of gastroparesis symptoms were particularly high among obese females with poor glycemic control and longer disease duration.

## Introduction

Gastroparesis or gastric stasis is a motility disorder resulting in delayed gastric emptying without evidence of mechanical obstruction [1,2]. The average time at which gastric contents empty into the small intestine is approximately 60 to 100 minutes, while patients with gastroparesis have emptying times ranging from 100 to >500 minutes [3].

Gastroparesis is often idiopathic but commonly related to diabetes mellitus (diabetic gastroparesis, DG) and post-gastric surgical conditions [4]. Gastroparesis is diagnosed in a patient with cardinal symptoms by demonstrating delayed gastric emptying that lacks any mechanical blockage. The manifestations of gastroparesis involve early satiety, postprandial fullness, bloating, nausea, and a visibly larger belly [5].

Gastric stasis is appealing to rising clinical and scientific attention in patients with type 2 diabetes mellitus (T2DM) [6]. Early features of delayed gastric emptying may present as poor glycemic control because gastroparesis lowers the insulin requirement and postprandial glucose peak [7].

An insignificant association had been previously reported between gastric emptying and the severity of gastroparesis-related symptoms [8,9]. In patients with functional dyspepsia, the delayed gastric emptying was related to excessive fullness after meals, bloating, nausea, and anorexia, whereas early satiety was related to impaired postprandial fundus relaxation, and abdominal discomfort was linked to hypersensitivity of the stomach to gastric distension [10,11].

The prevalence of DG varies widely. Several studies intended to determine the association between gender differences and the prevalence of gastroparesis among DM patients [12,13], and have established that comorbid conditions (age, alcohol ingestion, and cigarette smoking) drastically downgrade the quality of life in patients [14,15].

The early indicators of gastroparesis are the manifestations of many gastrointestinal disorders, and gastroenterologists regularly establish the given complication too late, as they are unable to detect any organic changes in the stomach. The predictors of DG remain inadequately studied [16], and severe gastroparesis has an undesirable prognosis. Whereas correction of gastric emptying with medication in patients with relative bradygastria influences the regularization of glucose metabolism and halts the volatile course of the primary disease [16].

This study intended to detect gastroparesis-related clinical symptoms early in T2DM patients, investigate the concomitant risk factors, and evaluate the prevalence.

## Materials and methods

Operational definitions

Gastroparesis is a prolonged motility disorder of delayed gastric emptying (100 to >500 minutes) that lacks any mechanical obstruction. The average time for gastric contents to empty into the small intestine is approximately 60 to 100 minutes. Mild (Grade 1) gastroparesis is categorized by well-resolved symptoms of dyspepsia. Compensated (Grade 2) gastroparesis is categorized by features that are managed with antiemetic and prokinetic medications. Gastric failure (Grade 3) gastroparesis does not respond to medication.

Adult-onset T2DM is characterized by hyperglycemia resulting from insulin resistance, insufficient insulin secretion, and excessive glucagon secretion.

Study design

This prospective cohort study was conducted at the Department of Medicine and Diabetes Outdoor Clinic of Sheikh Zayed Hospital, Rahim Yar Khan from February 13, 2022, to February 11, 2023.

The inclusion criterion included patients with T2DM who were receiving insulin therapy and/or in combination with oral antidiabetic drugs. Patients with gastrointestinal diseases, peptic ulcer disease, and previous history of upper gastrointestinal surgery were excluded from the research.

Data collection

After ethical research approval from Sheikh Zayed Medical College and Hospital (reference number: 126/IRB/SZMC/SZH), 175 patients satisfying the inclusion criteria were selected. The rationale of the study was described to patients and explicit consent was taken.

All patients received printed special questionnaire proforma. The demographic profile, severity of clinical features, disease duration, medications, concomitant risk factors, complications, and recent blood glucose and glycated hemoglobin (HbA1c) levels were collected and investigated. Body mass index (BMI (kg/m^2^) was estimated as weight in kg/height in m^2^. Blood glucose levels and HbA1C were measured using automatic analyzers.

The severity of DG was established using the disease-specific Patient Assessment of Gastrointestinal Disorders-Symptom Severity Index (PAGI-SYM) and the Gastroparesis Cardinal Symptom Index (GCSI) [17]. The symptoms were scored on a five-point scale (0 = absent, 1 = very mild, 2 = mild, 3 = moderate, 4 = severe, 5 = extremely severe). According to the PAGI-SYM, a score from 1 to 20 implies a very mild degree of gastroparesis, 21-40 signifies a mild degree of severity, 41-60 shows a moderate degree of severity, 61-80 score specifies severe gastroparesis, and 81-100 designates extremely severe gastroparesis.

The GCSI encompasses three PAGI-SYM subscales to determine important symptoms of gastroparesis, including postprandial symptoms/early satiety, bloating, and nausea/vomiting. The subscale postprandial fullness/early satiety assesses stomach fullness, inability to finish or feeling excessively full after a regular meal, and loss of appetite. The subscale bloating encompassed bloating and a visibly larger stomach or upper belly after meals. The subscale nausea/vomiting comprises nausea, retching, and vomiting. According to the GCSI severity, a score of 1-11 implies a mild degree, 12-22 shows a moderate degree of severity, 23-33 signifies a severe degree of gastroparesis, whereas more than 34 indicates an extremely severe degree of gastroparesis [18].

The severity of polyneuropathy was assessed using the Neurological Symptoms Score which verifies neurological symptoms and quantitative signs encompassed in the Neuropathy Disability Score [19]. Mild diabetic polyneuropathy with a score of five points; moderate with 5-13 points, whereas 14-19 points as severe diabetic polyneuropathy.

The stomach emptying function was calculated using the 13C-octanoate breath test (13C-OBT). The breath samples were evaluated using isotope-ratio infrared spectroscopy with the quantification of ^13^CO_2_ concentration. The half-time (Т½) of gastric emptying was assessed to confirm the gastric evacuation function. The normal reference range for Т½ is 40-75 minutes, Т½ <40 minutes shows gastric motility acceleration, Т½ of 75-95 minutes directs mild gastric motility deceleration, Т½ of 96-155 minutes signifies moderate gastric motility deceleration, Т½ >155 minutes suggests severe gastric motility deceleration [20].

Data analysis

Data analysis from questionnaires and special proforma was done using SPSS version 23 (IBM Corp., Armonk, NY, USA). Categorical variables are described using frequency (%). The mean values of age, gender, weight, height, BMI, systolic and diastolic blood pressure, diabetes duration, fasting plasma glucose, HbA1c, treatment types, and history of comorbidities were matched between gastroparesis and no gastroparesis groups. Relative risk factors were calculated with 95% confidence intervals. A p-value <0.05 was considered significant.

## Results

The study included 175 patients with T2DM who completed the questionnaires. The demographic and clinical characteristics are presented in Table 1. The disease duration of diabetes was 9.3 ± 0.5 years, the patient’s average age was 54.3 ± 0.8 years, and 63.8% of the patients were female. The mean BMI was 31.06 ± 5.1 kg/m2, the waist circumference was 96.6 ± 1.4 cm, and most patients used metformin (64.5%). The fasting glycemia was 169 ± 62.3 mg/dL, postprandial glycemia 186 ± 0.3 mg/dL, and HbA1C was 9.2% ± 4.1%. Hypertension affected 84 (48%) patients, 63 (36%) had hypercholesterolemia, and 28 (16%) had both. Other comorbidities were also recorded as follows: microvascular complications (retinopathy 32.3% and nephropathy 31.4%) and macrovascular complications (polyneuropathy and cardiovascular diseases 15.7%).

**Table 1:** Demographic and clinical characteristics of type 2 diabetes mellitus patients.

Demographic	Total, N (%)	Gastroparesis, N (%)	Diabetic gastroparesis, N (%)	P-value
N (%)	175 (100%)	10.9%	44%	
Age	54.3 ± 0.8	54.12 ± 14.5	53.39 ± 12.1	0.09
Gender				
Male	36.2%	10.4%	83.6%	0.08
Female	63.8%	12.9%	87.1%	0.06
Weight	76.8 ± 13.6	75.1 ± 11.6	77.04 ± 13.9	0.07
Height	160.6 ± 9.9	159.1 ± 8.42	160.7 ± 10	0.08
Body mass index	31.06 ± 5.1	30.32 ± 5.8	30.1 ± 5.5	0.06
Systolic blood pressure (mmHg)	142 ± 20.4	142 ± 17.8	142 ± 20.8	0.08
Diastolic blood pressure (mmHg)	80 ± 13.2	77 ± 12	81 ± 13.4	0.09
Duration of diabetes	9.3 ± 0.5	12.3 ± 5.4	9.96 ± 7.5	0.02
Fasting plasma glucose (mg/dL)	169 ± 62.3	211.71 ± 64	169.4 ± 56.1	0.003
Glycosylated hemoglobin (%)	9.2 ± 4.1	10.5 ± 2.2	8.89 ± 3.4	0.001
Type of treatment				
Metformin ± oral hypoglycemic drugs	113 (64.5%)	10.6%	89.4%	0.09
Insulin	62 (35.5%)	24.6%	75.4%	0.08
History of comorbid conditions				
Hypertension	48%	13%	87%	0.009
Hypercholesterolemia	36%	18%	82%	0.07

According to the results of performa, physical examination data, and laboratory investigations, mild DG was observed in 38 (21.7%) T2DM patients, moderate DG was noted in 30 (17.1%) patients, and severe DG symptoms were noticed in nine (5.2%) patients only. Thus, delayed gastric emptying was found in 44% of T2DM patients. The prevalence according to GCSI was 43% overall. The main manifestations were early satiety, stomach fullness, bloating, and nausea (45.1%, 44.5%, 38.3%, and 33.1%, respectively) (Table 2).

**Table 2:** Frequency of gastroparesis symptoms among type 2 diabetes mellitus patients (N = 175).

Gastroparesis symptoms	N (frequency %)
Nausea	58 (33.1)
Retching	25 (14.2)
Stomach fullness	78 (44.5)
Vomiting	7 (4)
Not able to finish a meal	79 (45.1)
Excessive fullness after meals	66 (37.7)
Loss of appetite	38 (21.7)
Bloating	67 (38.3)
Belly visibly larger	61 (34.8)

The severity of DG was established using the disease-specific PAGI-SYM and GCSI. The five-point scale of PAGI-SYM and four-degree severity scores of GCSI were completed. To predict delayed gastric emptying, there was a direct relationship between the special diabetic performa and relative risk factors of gastroparesis (Table 3).

**Table 3:** Relative risk factors of developing gastroparesis in patients with type 2 diabetes mellitus for different groups. GCSI = Gastroparesis Cardinal Symptom Index; PAGI-SYM = Patient Assessment of Gastrointestinal Disorders-Symptom Severity Index; RR = relative risk; CI = confidence interval; DM = diabetes mellitus; T2DM = type 2 diabetes mellitus

RR factors developing gastroparesis with disease-specific performa results in different Groups	Disease-specific questionnaires			
	GCSI points		PAGI-SYM points	
	RR	95% CI	RR	95% CI
Anamnestic data – a positive family history of DM	3.86	0.07–6.83	6.78	0.35–14.16
Concomitant cardiovascular diseases	2.74	1.20–4.28	6.57	2.99–10.16
Microvascular T2DM complications	3.71	2.18–5.25	9.05	5.49–12.62
Hypercholesterolemia	2.51	1.09–3.93	5.75	2.43–9.07

**Figure 1:**
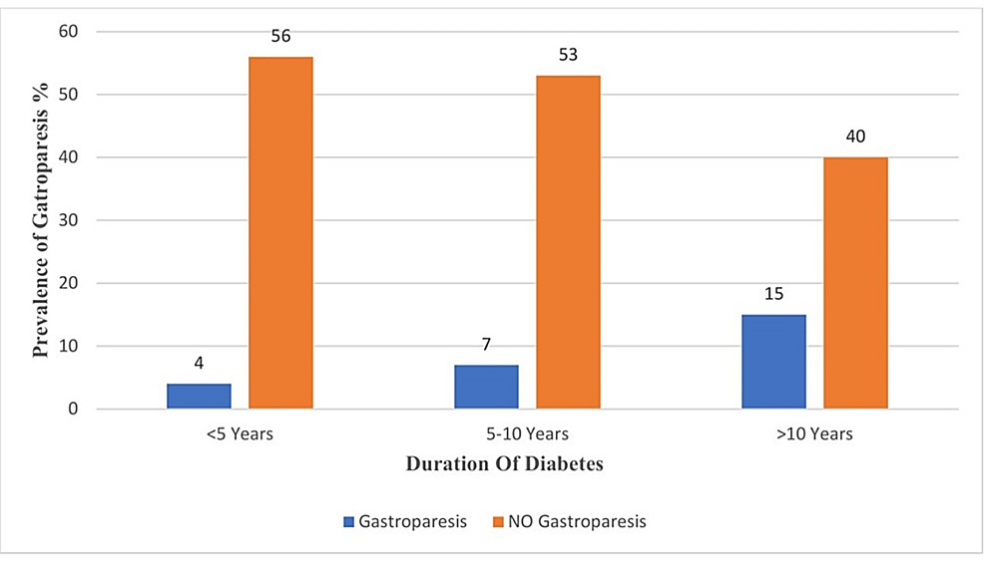
The prevalence of gastroparesis based on the duration of diabetes groups.

The prevalence of DG was found more in patients with greater than 10 years of diabetes duration than those with fewer than 10 years (Figure 1).

**Figure 2:**
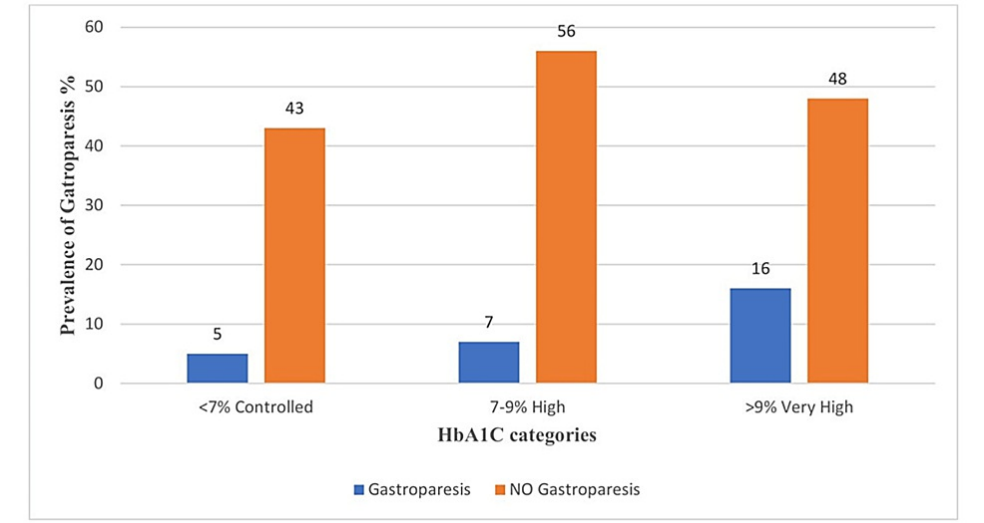
The prevalence of gastroparesis based on glycated hemoglobin categories.

Furthermore, the prevalence of DG was substantially greater in T2DM patients with >9% (very high) HbA1c than in patients with 7-9% (high) HbA1c or <7% (controlled) HbA1c (Figure 2).

DG symptoms were significantly correlated to disease duration of more than 10 years (p = 0.02), high HbA1c (p = 0.001), increased fasting blood glucose (p = 0.003), polyneuropathy, cigarette smoking, and comorbid conditions (p = 0.009). They included hypercholesterolemia, chronic microvascular complications, concomitant cardiovascular diseases, and a positive family history of diabetes. There was no relationship between the patient’s age, BMI, treatment types, and the degree of gastroparesis severity. Obesity and female gender appeared as noteworthy additional predictors of the presence of gastroparesis symptoms.

## Discussion

This is the first study to detect gastroparesis-related clinical symptoms early in the duration of the disease, investigate the concomitant risk factors, and evaluate the prevalence among Pakistanis with T2DM.

The prevalence of this study revealed that 44% of Pakistani T2DM patients have clinical gastroparesis-related symptoms based on disease-specific performa (PAGI-SYM and GCSI). A parallel estimation of 30% of T2DM patients having gastroparesis and up to 40% of T1DM was noted by Koch et al. [1]. It is similar to the approximated prevalence of 45.5% reported by Kostitska et al. [3] and 10.8% reported by Almogbel et al. [4], and these were analogous to prior estimations. These variants in the prevalence can possibly be associated with the demographic or clinical profile of the study population, diagnostic methods, and inclusion or exclusion criteria of gastroparesis patients. The microvascular complication of diabetic peripheral polyneuropathy was not a strong marker of DG as opposed to the report of Chuenyong et al. [5]. This suggested that polyneuropathy can predict the neuropathy of the stomach and should be regarded as a screening risk factor for gastroparesis.

The prevalence of DG in this study was drastically found in patients with a longer duration of T2DM. Patients with diabetes of more than 10 years duration had a higher prevalence than those with less than 10 years of disease duration (Figure 1), which was previously confirmed by similar studies [6,7]. Hyperglycemia with poor glycemic control was reported to be the underlying disease phenomenon linked to delayed gastric emptying [8,9].

The researchers suggested that cigarette smoking should be considered as the risk factor of polyneuropathy symptom progression, as noticed by Rodrigues et al. [7] and Shen et al. [8], which, in turn, is suggestive of gastroparesis prognostic factor. Obesity may worsen and participate in the progression of DG symptoms as suggested by Parkman et al. [10] and Jehangir et al. [11]. The prevalence and severity of gastroparesis symptoms are predominantly higher in uncontrolled T2DM with greater than 10 years of disease duration along with high BMI, as reported by Chedid et al. [12] and Dickman et al. [13].

This study showed that most patients who presented in diabetic clinics had poor glycemic control, high fasting plasma glucose, and higher HbA1C which contributed to delayed gastric emptying. The prevalence of DG was substantially greater in T2DM patients with >9 % (very high) HbA1c than in patients with 7-9% (high) HbA1c or <7% (controlled) HbA1c (Figure 2). A similar result suggesting a strong correlation between gastroparesis-related symptoms with higher HbA1C and hyperglycemia was reported by Parkman et al. [14], Sharma et al. [15], and Kim et al. [16]. This showed that good glycemic control in T2DM patients helps recover gastroparesis-related symptoms and complications [17-20]. Whereas previously, several studies did not establish a reliable correlation of gastroparesis with BMI, fasting blood glucose, and HbA1C; however, concomitant cardiovascular diseases and microvascular diabetes complications have revealed clear associations with DG, as noticed by Moors et al. [18], Revicki et al. [19], and Navas et al. [21].

A correlation was reported by Nelson et al. [22] between DG incidence with both age and insufficient glycemic control, while this study along with previous studies did not document such dependence [23-25]. However, the results coincide with the studies of Friedenberg et al. [20] and Camilleri et al. [25], agreeing that the female gender seemed a significant predictor of DG [25-27]. Estrogen level changes might explain this variation [28,29]. Moreover, women experienced most functional gastrointestinal disorders in contrast to men [29]. Cardiovascular diseases, hypertension, and retinopathy were detected more often in patients with DG due to poor glycemic control [26-29].

Gastric scintigraphy is the gold standard technique for assessing gastric emptying [30]. This study relied on special performa with GCSI severity score, which helped stratify gastroparesis-related symptoms and treatment outcomes.

In T2DM patients, longer duration of diabetes, higher HbA1c, comorbidities, and microvascular and macrovascular complications have been recognized as major risk factors to detect DG early. Therefore, early detection of DG prevented the progression of the disease along with laboratory parameters.

## Conclusions

Gastric emptying is significant in the pathogenesis of gastroparesis-related symptoms. Diabetes mellitus duration of more than 10 years, poor glycemic control with hyperglycemia, high HbA1C, polyneuropathy, and cigarette smoking must be considered as predictors for early detection and risk factors for the advancement of gastroparesis in T2DM. Gastroparesis-related common symptoms of early satiety, bloating, and stomach fullness were considerably linked to the additional risk factors of hypercholesteremia, chronic microvascular complications, concomitant cardiovascular diseases, and a positive family history of DM. There was no relationship between the patient’s BMI, age, types of treatment, and the degree of gastroparesis severity. The prevalence and severity of gastroparesis symptoms are particularly high among obese females with poor glycemic control and longer disease duration.

Therefore, routine use of disease-specific questionnaires for patients will allow physicians to detect risk factors early, prevent gastrointestinal complications, and help improve treatment in patients with T2DM.
